# Supplementary material for: A gene-expression screen identifies a non-toxic sumoylation inhibitor that mimics SUMO-less human LRH-1 in liver
Source: eLife. 2015 Dec 11;4:e09003. doi: 10.7554/eLife.09003 (PMC4749390; doi:10.7554/eLife.09003)
Supplement: Supplementary file 1. — DOI: http://dx.doi.org/10.7554/eLife.09003.022 [file elife-09003-supp1.zip › Supplementary file 1.pdf]

Supplementary File 1

Supplementary Table S1. RT-qPCR Primer Sequences

Name	Accession	Forward 5'-3'	Reverse 5'-3'
Adipoq	NM_009605	CGACACCAAAAGGGCTCAG	TGTAGTAACGTCATCTTCGGCA
APOC3	NM_000040	CCCCGGGTACTCCTTGTTG	TTGGTGGCGTGCTTCATGTA
CYP11A1	NM_000781	GGGTCGCCTATCACCAGTATT	GCTGCCGACTTCTTCAACAG
Cyp8b1	NM_010012	GAGCCCACAGCCTTCAAGTAT	TCTTCTTGCCCGACTTGTAGA
eGFP		GAAGGGCATCGACTTCAAGG	TTCTGCTTGTCGGCCATGATA
Gapdh	NM_008084	GGCGCGCGTCATCAG	TGACCAGGCGCCCAATAC
Gli2	NM_001081125	TGGTGATCACTCGAGTTTGTT	CTGGGAGAGATTGTGGAGAAG
MUC1	NM_182741	ACCCATGGGCGCTATGTG	ACCATTACCTGCAGAAACCTT
NR5A2 (hLRH-1)	NM_205860	CAGAGAAAGCGTTGTCCTTACTG	TTATTCCTTCCTCCACGCATT
Nr5a2 (mLrh-1)	NM_139051	CGTCTGTCTCAAGTTCCTCATCCT	TCCTTTACGAGGCTGTGGTTGT
PFKFB3	NM_004566	GAAGCAGTACAGCTCCTACAACTTC	ACATTGCTTCCGGACTTTCA
SERPINE1	NM_000602	CGCCAGAGCAGGACGAA	CATCTGCATCCTGAAGTTCTCA
Shh	NM_009170	GAGGTGCAAAGACAAGTTAAATGC	CGGTCACTCGCAGCTTCAC
TBP	NM_003194	CGAATATAATCCCAAGCGGTTT	TGGTTCGTGGCTCTCTTATCC
