# Supplementary material for: A gene-expression screen identifies a non-toxic sumoylation inhibitor that mimics SUMO-less human LRH-1 in liver
Source: eLife. 2015 Dec 11;4:e09003. doi: 10.7554/eLife.09003 (PMC4749390; doi:10.7554/eLife.09003)
Supplement: Supplementary file 2. — DOI: http://dx.doi.org/10.7554/eLife.09003.023 [file elife-09003-supp2.zip › Supplementary file 2.pdf]

Supplementary File 2. Summary of Small Molecule Screening Data

Category	Parameter	Description
Assay	Type of assay	Gene expression-based Screen Assay
	Target	LRH-1 sumo-sensitive genes (*APOC3* and *MUC1*)
	Primary measurement	mRNA transcript
	Key reagents	SYBR
	Assay protocol	Described in Material and Methods
	Additional comments	LRH-1 SUMO-sensitive genes were identified by microarray profiling in JEG3 hLRH-1 cells<br>TBP (house keeping gene) expression was used as an internal control
Library	Library size	1600
	Library composition	Biologically active FDA approved compound
	Source	Micro Source's Pharmakon Collection
	Additional comments	None
Screen	Format	384 well plates
	Concentration(s) tested	10 µM compound and 0.1% DMSO
	Plate controls	DMSO treated cells
	Reagent/ compound dispensing system	Biomek FXP/ 50nL pin-tool
	Detection instrument and software	ABI 7900HT/SDSv2.3
	Assay validation/QC	CV < 20% with DMSO control (N=70)
	Correction factors	Ave of Z-score ± 2SD
	Normalization	TBP as internal control<br>Pooled DMSO – treated sample as external control
	Additional comments	None
Post-HTS analysis	Hit criteria	Increase or decrease in *APOC3* and *MUC1*
	Hit rate	1 validation of final progress out of 1600
	Additional assay(s)	Change in FL-hLRH-1 protein in vitro sumoylation<br>Change in hLRH-1 expressed in JEG3, HepG2 and primary humanized hepatocytes<br>Gene expression Assay of Other Genes
	Confirmation of hit purity and structure	ASC grade
	Additional comments	None
