# Supplementary material for: A gene-expression screen identifies a non-toxic sumoylation inhibitor that mimics SUMO-less human LRH-1 in liver
Source: eLife. 2015 Dec 11;4:e09003. doi: 10.7554/eLife.09003 (PMC4749390; doi:10.7554/eLife.09003)
Supplement: Supplementary file 3. — DOI: http://dx.doi.org/10.7554/eLife.09003.024 [file elife-09003-supp3.zip › Supplementary file 3.pdf]

Supplementary File 3. Drug Acquisition Information

Name	Supplier	Catalog No
2-D08	Transfer from Dr. J.S. Schneekloth	
Ginkgolic Acid (C15:1)	SANTA CRUZ	SC-235249
Colforsin (NKH 477)	Cayman Chemical	11214
Ouabain	TOCRIS Bioscience	630-60-4
Tannic acid	Sigma-Aldrich	T0125
Taxol (Paclitaxel)	Sigma-Aldrich	T1912
Trifluridine	FISHER SCIENTIFIC	505910
Vincristine	Cayman Chemical	11764
